# Deep learning based diagnostic quality assessment of choroidal OCT features with expert-evaluated explainability

**DOI:** 10.1038/s41598-023-28512-4

**Published:** 2023-01-28

**Authors:** S. P. Koidala, S. R. Manne, K. Ozimba, M. A. Rasheed, S. B. Bashar, M. N. Ibrahim, A. Selvam, J. A. Sahel, J. Chhablani, S. Jana, K. K. Vupparaboina

**Affiliations:** 1 Indian Institute of Technology Hyderabad, Kandi, 502284 India; 2 University of Alabama-Birmingham School of Medicine, 35233 Birmingham, AL USA; 3 School of Optometry and Vision Science, University of Waterloo, Waterloo, N2L 3G1 Canada; 4 Manzor Alam Opticals, Murshidabad, 742236 India; 5 University of Pittsburgh School of Medicine, Pittsburgh, PA 15213 USA

**Keywords:** Imaging, Diagnosis, Tomography, Image processing, Machine learning, Eye diseases

## Abstract

Various vision-threatening eye diseases including age-related macular degeneration (AMD) and central serous chorioretinopathy (CSCR) are caused due to the dysfunctions manifested in the highly vascular choroid layer of the posterior segment of the eye. In the current clinical practice, screening choroidal structural changes is widely based on optical coherence tomography (OCT) images. Accordingly, to assist clinicians, several automated choroidal biomarker detection methods using OCT images are developed. However, the performance of these algorithms is largely constrained by the quality of the OCT scan. Consequently, determining the quality of choroidal features in OCT scans is significant in building standardized quantification tools and hence constitutes our main objective. This study includes a dataset of 1593 good and 2581 bad quality Spectralis OCT images graded by an expert. Noting the efficacy of deep-learning (DL) in medical image analysis, we propose to train three state-of-the-art DL models: ResNet18, EfficientNet-B0 and EfficientNet-B3 to detect the quality of OCT images. The choice of these models was inspired by their ability to preserve the salient features across all the layers without information loss. To evaluate the attention of DL models on the choroid, we introduced color transparency maps (CTMs) based on GradCAM explanations. Further, we proposed two subjective grading scores: overall choroid coverage (OCC) and choroid coverage in the visible region(CCVR) based on CTMs to objectively correlate visual explanations vis-à-vis DL model attentions. We observed that the average accuracy and F-scores for the three DL models are greater than 96%. Further, the OCC and CCVR scores achieved for the three DL models under consideration substantiate that they mostly focus on the choroid layer in making the decision. In particular, of the three DL models, EfficientNet-B3 is in close agreement with the clinician’s inference. The proposed DL-based framework demonstrated high detection accuracy as well as attention on the choroid layer, where EfficientNet-B3 reported superior performance. Our work assumes significance in bench-marking the automated choroid biomarker detection tools and facilitating high-throughput screening. Further, the methods proposed in this work can be adopted for evaluating the attention of DL-based approaches developed for other region-specific quality assessment tasks.

## Introduction

**Figure 1:**
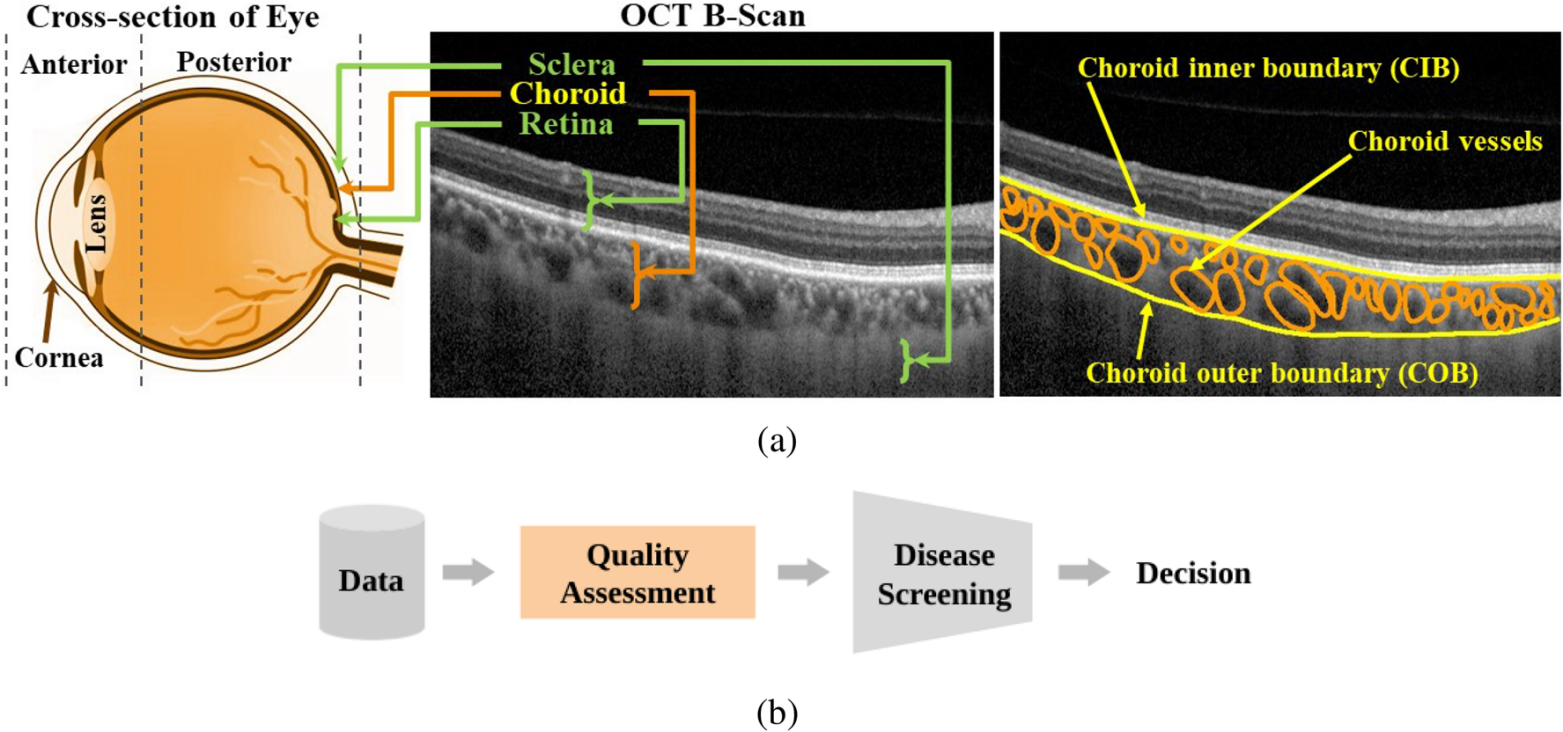
(**a**) Sagittal cross-section of the eyes (*Left*); Sample OCT cross-sectional image (B-scan) depicting posterior segment layers including retina, choroid, and sclera (*Middle*); and OCT B-scan depicting choroidal boundaries and vessels (*Right*), and (**b**) Desired disease screening pipeline with quality assessment as an essential step.

Many eye diseases that lead to permanent vision impairment originates due to structural changes in the choroid, a vascular layer located between retinal and scleral layers of the posterior segment of the eye (see Fig. 1a). Some of the prevalent diseases associated with choroid include central serous chorioretinopathy (CSCR), age-related macular degeneration (AMD) and macular edema^1–3^. In deed, choroid is responsible for the health of the retina and the other structures of the eye as it supplies oxygen and nutrient to them. Accordingly, detection of structural changes in the choroid play a crucial role in disease diagnosis and management. In the current clinical practice, ubiquitous optical coherence tomography (OCT) imaging has enabled clinicians with in-vivo substructural visualization of retina, choroid and scleral layers^4–7^. A sample OCT B-scan (cross-sectional) image is depicted in Fig. 1a. In particular, OCT imaging facilitates clinicians to screen the choroid both qualitatively and quantitatively^8–11^. Specifically, clinicians seek to quantify various biomarkers including choroidal thickness (CT), choroidal volume (CV) and choroidal vascularity index (CVI) based on OCT images^12–14^. Accurate quantification of such clinical determinants determine the diagnostic accuracy. In the recent past, several attempts have been made towards development of automated tools for accurate detection of choroidal biomarkers^13–15^. In particular, almost all the automated algorithms reported presume that the datasets considered are of good quality OCT images. However, in practice, datasets may be of varied quality and the performance of those algorithms is majorly constrained by the input image quality^16–19^.

For instance, often times algorithms developed based on good quality B-scans may end up encountering bad quality images and produce a spurious measurement. Specifically, the performance of an algorithm developed for choroidal biomarker detection majorly depends on the quality of the choroidal features such as (i) contrast between luminal (vessel) and stromal (non-vessel) region, (ii) contrast between choroid and retina (or sclera), (iii) speckle-noise due to coherence of light and (iv) signal attenuation due to retinal structural changes. Against this backdrop, it is imperative to assess the choroid quality of the OCT scans determining their clinical gradability to facilitate automated disease screening and prognosis (as shown in Fig. 1b). Such a quality assessment tool may also enable clinicians with high-throughput screening in resource-constraint scenarios.

Image quality assessment (IQA) is a well-posed problem for natural images especially in the context of transmission and broadcasting^20^. There has been a huge leap forward in developing accurate methods, both formula- and learning-based, to find the quality of natural images^21,22^. However, attempts at IQA of medical images i.e, diagnostic quality assessment (DQA), especially in relation to ophthalmological disease diagnosis are very limited^23,24^. More specifically, majority of attempts were directed towards DQA of fundus photography (FP) images focusing on accurate detection of specific diseases such as diabetic retinopathy (DR)^25^. Further, DQA of FPs has been addressed using traditional features^26^, wavelet-based deep scattering features^27^ and deep learning (DL)-based methods^28,29^. On the other hand, DQA of OCT images is relatively less explored. Very few attempts were made at OCT image DQA in relation to disease detection. Early attempts in assessing IQA of OCT images were based on traditional approaches that use image histograms^30,31^, intensity histogram decomposition model^32^, and complex wavelet based local binary pattern features^33^. Further, very limited works have been reported using DL-based models for IQA of OCT A-scans and B-scans^34,35^. Recently, an attempt using transfer learning was made for multi-class IQA of OCT images in distinguishing images with signal occlusion and off-center^36^.

Unfortunately, there are not many studies on both FP and OCT at determining quality of image with attention to specific structure such as choroid layer. Recently, a method has been reported for detecting the region-specific quality of FP focusing on visibility and clarity of regions such as optic disc and fovea^28^. However, DQA of OCT (OCT-DQA) images is not explored much in this direction. Consequently, there are no attempts to assess the quality of choroid region in OCT images which may enable development of standardized choroidal biomarker detection tools. In response, we propose to develop an approach to determine the DQA of OCT images to enable the accurate detection of choroidal biomarkers. Specifically, noting the performance of the DL-based learning methods over the traditional methods, we propose to train three state-of-the-art DL-models, namely, ResNet18, EfficientNet-B0 and EfficientNet-B3 towards OCT-DQA^37,38^. Further, to understand how these models detect the DQ of OCT with attention on choroidal features, we employ recently introduced concepts of explainability for DL models such as gradient weighted class activation maps (Grad-CAM)^39^. The summary of the proposed approach and contributions are enumerated in the following.

- Attempted choroid region-specific diagnostic quality assessment of OCT images.
- Trained three state-of-the-art DL models, namely, ResNet18 and EfficientNet-B0 & -B3 for OCT-DQA and demonstrated performance over 96% detection accuracy.
- Created an OCT dataset of 4174 B-scan images graded by an expert for binary classification (good/bad) based on the quality of the choroid layer.
- Introduced color transparency maps (CTM) based on Grad-CAM that aid clinicians in visualizing the relevant regions of the model’s decision
- Proposed two grading scores based on transparency maps, namely, overall choroid coverage (OCC) and choroid coverage within visibility region (CCVR), for evaluating the attention of DL models on the choroid layer.
- Demonstrated the importance of choroid quality assessment in screening chorioretinal diseases.

## Results

We now proceed to evaluate the performance of the three models under consideration. First, we compare the performance indices obtained by the three models vis-à-vis that of other state-of-the-art methods. Subsequently, we discuss the CTM visualizations obtained based on Grad-CAM followed by a pilot study on the impact of choroid quality assessment in chorioretinal disease screening.

**Table 1:** Performance indices over 5-fold cross-validation (%).

Model	AUC	Accuracy$^\ddagger$	Precision$^\ddagger$	Recall$^\ddagger$	F1-Score$^\ddagger$
BRISQUE^40^	$0.648 \pm 0.012$	$62.73 \pm 1.59$	$52.39 \pm 4.31$	$25.38 \pm 3.09$	$34.14 \pm 3.49$
NBIQA^41^	$0.741 \pm 0.021$	$71.14 \pm 1.15$	$67.56 \pm 2.31$	$47.03 \pm 2.50$	$55.41 \pm 2.01$
ScatNet^26^	$0.939 \pm 0.013$	$87.54 \pm 1.88$	$84.41 \pm 3.27$	$82.74 \pm 2.22$	$83.54 \pm 2.36$
ResNet18	$0.997 \pm 0.002$	$97.69 \pm 0.62$	$\mathbf {98.66 \pm 0.38}$	$96.72 \pm 1.05$	$97.67 \pm 0.64$
EfficientNet-B0	$0.995 \pm 0.002$	$96.99 \pm 0.59$	$97.82 \pm 0.32$	$96.12 \pm 1.08$	$96.96 \pm 0.61$
EfficientNet-B3	$\mathbf {0.997 \pm 0.001}$	$\mathbf {97.92 \pm 0.41}$	$98.65 \pm 0.60$	$\mathbf {97.17 \pm 0.57}$	$\mathbf {97.90 \pm 0.41}$

$^\ddagger$ Values recorded at operating point with maximum accuracy

Significant values are in bold.

### OCT choroid quality assessment

The performance indices obtained from various models are presented in Table 1. Clearly, the proposed DL-based models: ResNet18, ENet-B0, and ENet-B3 perform significantly better than previously reported natural IQA-metric-based methods including BRISQUE^40^, NBIQA^41^, and ScatNets^26^. In particular, among natural IQA-metric-based methods, BRISQUE and NBIQA, respectively, achieved mean accuracy values of 62.73 and 71.14% which is relatively poor vis-à-vis corresponding accuracy value 87.54% obtained by ScanNet based approach. This probably can be attributed to the structure-preserving nature of the ScatNets. In contrast, the mean accuracy values of ResNet18, ENet-B0 and ENet-B3 are observed to be 97.69, 96.99 and 97.92%, respectively, demonstrating significantly high performance against previous methods. Among the DL-Methods under consideration, ENet-B3 performed marginally better than ResNet18 and ENet-B0, especially in terms of variability (0.41%). This observation is consistent with other metrics including F-score, AUC, recall. ResNet18 is marginally better in terms of precision.

**Figure 2:**
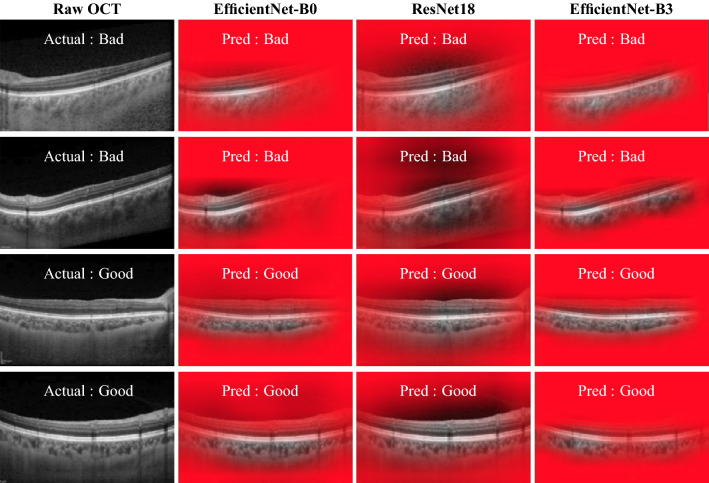
Representative images of OCT images with CTMs corresponding to all three models. While top two rows correspond to bad quality OCT image, the bottom two rows correspond to good quality OCT image.

### Visual explanations

We now proceed to evaluate the DL-Models based on the CTMs to understand their focus areas in performing the detection task. Figure 2 depicts representative images for good and bad quality OCT scans with CTMs obtained for all the three models under consideration. Notice that, for all the representative images, actual labels match the predicted labels of all three models. More interestingly, across all three models, the visibility region in the CTMs that contributes to the model’s decision is observed to be around the choroid. Recalling our primary task of discriminating the OCT images based on the quality of the choroidal features, the visual explanations of the DL models considered in this work strongly correlate with the desired outcome. However, among the three models, ENet-B3 appears to be largely confined to the choroid whereas the other models appear to be spanning into other layers including the retina and the sclera.

**Table 2:** Mean scores of subjective grading performed on CTMs.

		OCC (%)			CCVR (%)			|OCC-CCVR| (%)		
		ENet-B0	ResNet18	ENet-B3	ENet-B0	ResNet18	ENet-B3	ENet-B0	ResNet18	ENet-B3
Healthy	*Good*	66.50	72.50	74.67	37.50	43.00	56.33	29.00	31.83	24.33
	*Bad*	83.67	78.16	82.67	60.17	56.00	67.67	24.83	23.50	15.00
Diseased	*Good*	63.50	70.50	73.67	36.33	45.5	50.00	28.17	25.33	27.00
	*Bad*	80.67	75.33	73.83	67.33	57.33	70.33	17.67	21.67	15.17
Overall		73.58	74.12	**76.21**	50.33	50.46	**61.08**	24.92	25.58	**20.37**

Significant values are in bold.

To corroborate the same, we now move towards analyzing subjective grading performed on CTMs. As mentioned earlier, we obtained subjective scores OCC and CCVR on a subset of 180 images (60 per model) by two masked observers. Next, we computed the Bland-Altman correlation between both the observers across OCC and CCVR. Encouragingly, the correlation between the respective OCC and CCVR scores obtained by both the graders is observed to be 97.32 and 94.61%, indicating good reliability of the subjective scores. Noting the high correlation between the graders, we considered average values of scores obtained by both the observers for further analysis on OCC and CCVR. To perform comprehensive evaluation, we computed the mean OCC and CCVR values for overall and sub-groups (Healthy-Good, Healthy-Bad, Diseased-Good, & Diseased-Bad) for all the three models under consideration (see Table 2). Further, we have also obtained absolute difference between OCC and CCVR values which measures the agreement between the both measures. Ideally, we desire high OCC and CCVR values and a low |OCC-CCVR| value.

For the three DL-models: ENet-B0, ResNet and ENet-B3, the overall mean OCC scores achieved are observed to be 73.58, 74.12 and 76.21% while the mean CCVR scores achieved are observed to be 50.33, 50.45, 61.08%, respectively (see Table 2). In comparison, both OCC and CCVR scores are high for ENet-B3 buttressing the qualitative observation made earlier on CTMs. Subsequently, the overall |OCC-CCVR| values for ENet-B0, ResNet and ENet-B3 models are observed to be 24.92, 25.58 and 20.37%, respectively, which also corroborates the ENet-B3’s relative performance efficacy.

Further, the mean OCC and CCVR scores for sub-groups indicate that good-quality (for Healthy and Diseased) images achieved low OCC and CCVR values for all three models while bad-quality (for Healthy and Diseased) images achieved high OCC and CCVR scores for all three models. The low scores corresponding to the diseased good-quality images may be because of the possible model’s attention only on depleted choroidal regions. Overall, ENet-B3 achieved high CCVR scores for all sub-groups and high OCC for healthy-good and diseased-good cases. However, |OCC-CCVR| values indicate that ENet-B3 is performing better. Notice, although for Diseased-Good case, ResNet18 is marginally better than ENet-B3, the mean OCC and CCVR values are high for ENet-B3 indicating its superiority over ResNet18.

**Figure 3:**
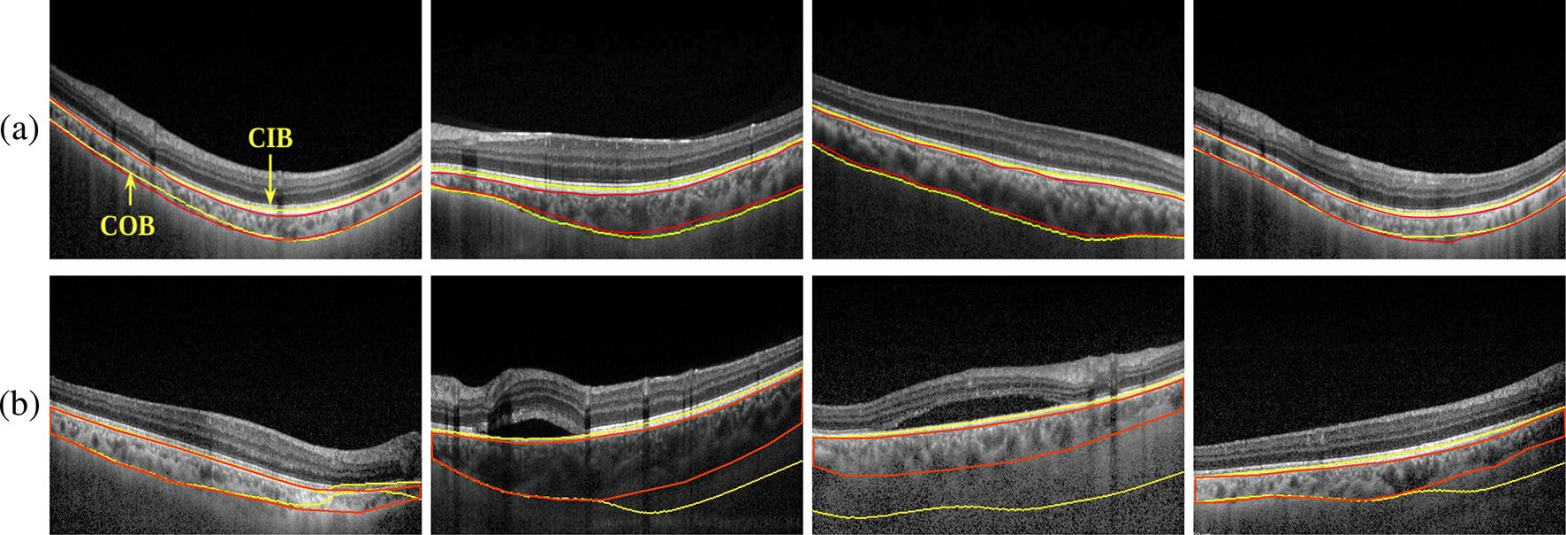
Choroid boundary (CIB & COB) detection on OCT images via both manual(red) and algorithm(yellow) with : (**a**) good quality, and (**b**) bad quality.

Finally, we investigate our hypothesis on the importance of choroid quality assessment in OCT images by considering a practical scenario. Specifically, we consider an automated tool for detecting choroidal inner boundary (CIB) and choroid outer boundary (COB)^42^, a primary step in screening or quantification of chorioretinal diseases. As a pilot study, we randomly selected few OCT images from both good and bad quality of our dataset, and obtained manual annotations of choroid boundaries by expert. Next, we pass the same set of images through the choroid detection tool^42^. As anticipated, on good quality images, both CIB and COB delineations by the automated tool are in agreement with the corresponding manual annotations (see Fig. 3a) while on bad quality OCT images, COB delineations by the automated deviated significantly from the corresponding manual ones (as shown in Fig. 3b), buttressing the need for a quality assessment tool as preprocessing step in the choroidal biomarker quantification pipeline (Fig. 1b).

## Discussion

In this paper, we attempted a DL-based quality assessment of the choroid layer in OCT images. Specifically, we examined three state-of-the-art models ResNet18, ENet-B0 and ENet-B3, and demonstrated their efficacy. In particular, all three models exhibited high performance with more than 96% accuracy and F1-score which is observed to be a significant leap vis-à-vis the performance of other IQA methods. We observed that ENet-B3 achieved marginally better performance which probably can be attributed to its higher input image size and depth. Further, we obtained novel color transparency maps a.k.a visual explanation maps to evaluate the models for their attention on choroidal features. Specifically, the mean subjective grading scores of overall choroid coverage and choroid visible region are observed to be high for ENet-B3.

The proposed work assumes significance in (i) standardizing the OCT image quality for automated choroid biomarker quantification tools. To this end, we plan to evaluate methods reported by our group^13,43^; (ii) enabling clinicians to identify clinically gradable images from years of retrospective data available in the tertiary centers like UPMC; (iii) facilitating clinicians in accurate and high throughput screening at tertiary centers and (iv) teleophthalmology based on portable OCT imaging. Further, the proposed methods including CTMs and grading scores (OCC and CCVR) can be adopted in evaluating the attention of DL-based tools developed for other region-specific quality assessment problems.

We envisage making the framework more robust by improving the data preparation and training. Accordingly, to improve the data, we plan to build a robust and large database of OCT images graded by multiple observers. Further, we plan to extend the current binary (good/bad) classification framework to multi-class classification defined based on multiple levels of quality including good, bad and usable.

In this work, as the selected DL models achieved satisfactory performance we did not get a chance to explore any architectural improvements of the DL models. However, we plan to modify the DL model architectures in the future work involving our modified database as alluded earlier. Further, we also plan to examine other recently reported DL-based methods including vision transformers (ViT) that are optimized to yield higher accuracies under resource-constrained settings^44^.

## Methods

This was a retrospective study conducted at University of Pittsburgh Medical School, USA.The study was approved by the institutional review board of the University of Pittsburgh Medical School. Informed consent was obtained from all participants to include their retrospective data in the study. All the methods adhered to tenets of the Declaration of Helsinki. All the subjects underwent optical coherence tomography (OCT) examination of the posterior pole of the eye. In particular, the OCT images were acquired using Heidelberg Retina Angiograph (HRA) Spectralis OCT machine. The axial and transverse scanning resolution was 7 and 14 $\mu$m, respectively. Further, the scanning speed of the Spectralis OCT device was 40,000 amplitude scans (A-scans) per second. Each B-scan captured is an average of 25 frames scanned. Overall, we have collected 1094 healthy and 3080 diseased B-scans.

**Figure 4:**
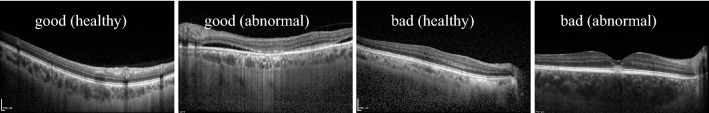
Representative OCT images annotated based on choroid quality by expert.

*Data annotation* The images were graded subjectively into two classes ‘good’ and ‘bad’ by a trained expert. Various parameters including visibility of the choroid, contrast between choroidal luminal (vessel) and stromal (regions), contrast between the choroid and scleral especially at choroid sclera interface (choroid outer boundary, COB) were considered while grading. After grading, we obtained 1593 good quality images (of which 488 are healthy and 1105 are diseased) and 2581 bad quality images (606 are healthy and 1975 are diseased). Figure 4, gives illustrative OCT images with both good quality and bad quality graded by the expert.

**Figure 5:**
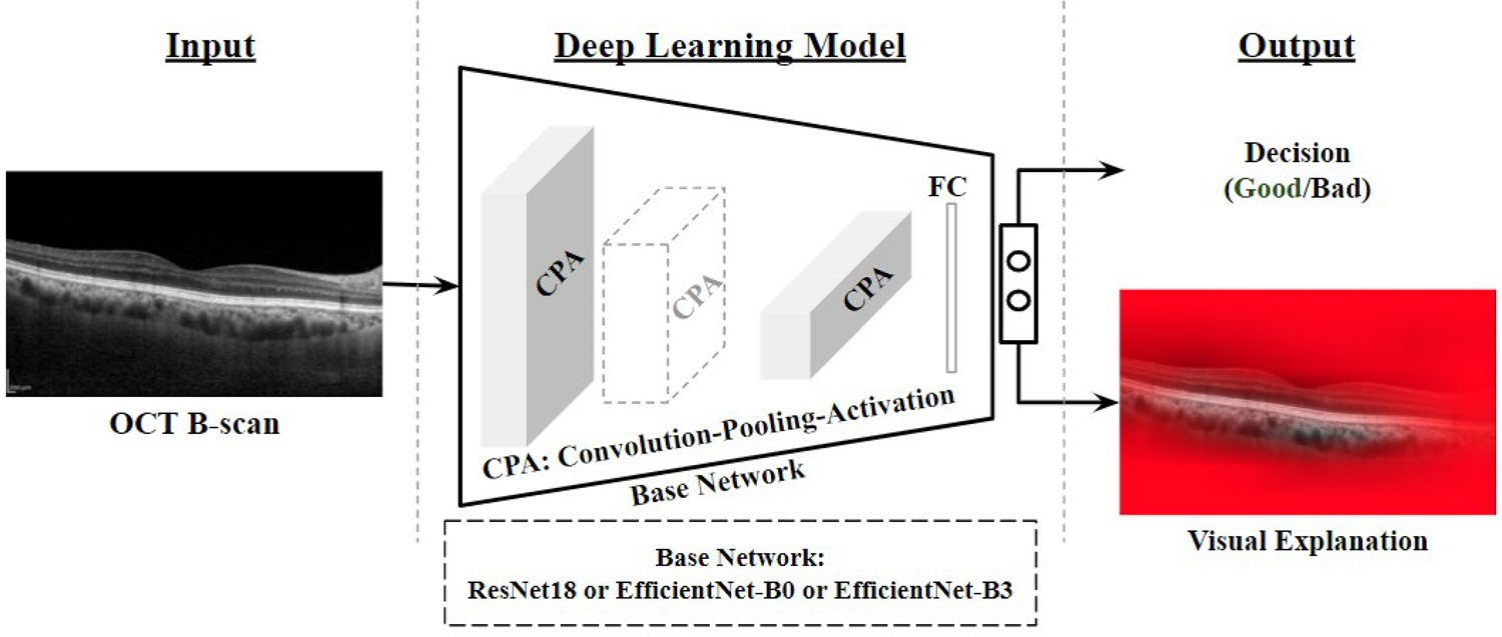
Schematic of the proposed pipeline for assessing quality of an OCT image.

In the proposed workflow, as outlined in Fig. 5, we seek to investigate the efficacy of deep-learning features in distinguishing the choroidal quality of OCT images. A detailed description on DL variants (EfficientNet and ResNet), Grad-CAM explanations and evaluation criteria are presented in the following subsections.

### Deep learning approach

Deep learning (DL) models attempt to perform image classification by employing convolution neural networks (CNN) to extract features that mimic human perception. To develop an efficient DL model, the crux lies in the optimal choice of the design parameters including input image resolution, the number of layers (depth), and the number of filters in each layer (width). The depth and the width determine the respective ability to learn the rich and complex features while the input resolution determines the ability to learn the fine-grained features^38^. Accordingly, several task-specific architectures have been developed with a trade-off among aforesaid design parameters. The last decade has witnessed tremendous breakthroughs in DL in the context of image classification where various models achieved near-perfect detection performance. In recent times, the two popular state-of-the-art DL models, namely ResNet (residual networks) and EfficientNet, trained on large public datasets of natural images have become the ubiquitous choice for transfer learning^37,38^. In particular, these models are known to preserve the salient features of the images across all the layers without information loss. Further, they train on a relatively less number of parameters when compared to other DL models. On the other hand, there have been efforts toward making the DL models explainable i.e, to understand the attention of DL models while learning the features. In particular, the explainability of the DL model may facilitate us to understand its agreement with human perception. Such explainability may be crucial in applications including disease screening based on images^45,46^. To this end, the recently proposed Grad-CAM visualization has been widely accepted to depict DL model attention map. Against this background, we adopt the aforementioned pretrained models, ResNet and EfficientNet, to detect the quality of choroidal features in OCT images. In particular, we consider ResNet/EfficientNet as the base network (initialized with pretrained ImageNet-weights) and replace the output layer with a binary classification head to suit the current application. The modified architectures are then trained on the OCT dataset at hand. Further, we investigate their performance based on Grad-CAM visualizations to understand their agreement with the clinician’s decision making. The details of the proposed methodology in connection to ResNet and EfficientNet architectures as well as Grad-CAM visualization are described in the following subsections.

**Figure 6:**
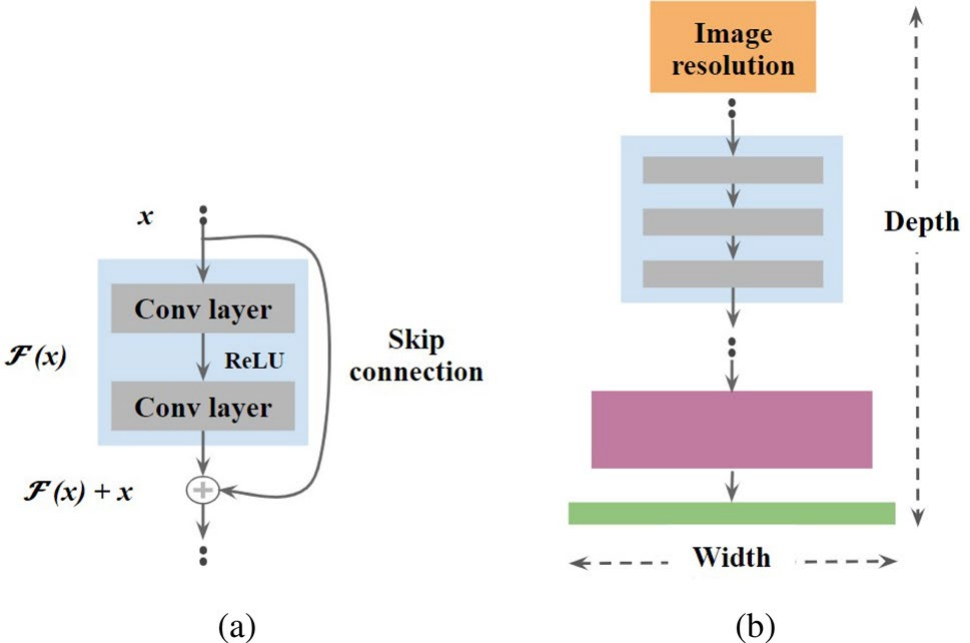
Frameworks in DL models: (**a**) Residual block; (**b**) Compound scaling.

**Table 3:** Implementation details of both EfficientNet and ResNet models

	Input	Learning rate	Epochs	Parameters	Loss
ResNet18	(224, 224)	0.0001	35	11.8*M*	Cross Entropy
ENet-B0	(224, 224)	0.0001	65	5.3*M*	Cross Entropy
ENet-B3	(300, 300)	0.0001	35	12*M*	Cross Entropy

#### ResNet

In feed-forward DL models, as the number of layers (depth) increase, the amount of information about the input (or gradients while backpropagation) may vanish as one approaches the final layers (or initial layers), and hence pose difficulty in the training process. To overcome this, a residual learning framework^37^ was proposed by introducing skip-connections between layers of network. In particular, as shown in Fig. 6a, the skip connections are introduced between each residual block *F*(*x*) which sequentially performs $3\times 3$ convolution, rectified linear unit (ReLU) activation, and another $3\times 3$ convolution operations. As a result, these skip connections not only allow the instances of previous layers in the feed-forward path, but also ensure that the gradients are always greater than one. Further, the number of such residual blocks determines the complexity of the model. In this paper, we consider the ResNet18 variant that takes the input of size $224\times 224$ with 18 layers and has approximately 11.8*M* parameters^37^. More details of the model are provided in Table 3.

#### EfficientNet

On the other hand, the EfficientNet employs compound scaling of the three aforesaid design parameters (image resolution, depth and width), and caters to practical resource constraints while maintaining model efficiency^38^. The original variant EfficientNet-B0 (ENet-B0) considers a baseline architecture MobileNetV1^47^, and performs compound scaling to optimize the three design parameters to meet the computational resource constraint (Fig. 6b). In particular, ENet-B0 takes images of resolution $224\times 224$ at the input layer and consists of a total of 237 layers with 5.3*M* parameters. The subsequent variants ENet-B1,..., ENet-B7 take higher resolutions at the input which resulted in a respective increase in complexity. For instance, ENet-B3 takes images of size $300\times 300$ at the input and has 384 layers, while ENet-B7 takes images of size $600\times 600$ at the input and has 813 layers^38^. However, an increase in complexity demands higher data and resources to train. Acknowledging this, we examined two Efficient variants including ENet-B0 and ENet-B3. Table 3 presents the design parameters of both the variants.

### Evaluation methods

#### Performance measures

We consider the ubiquitous metrics such as accuracy, precision, recall and F1-score for evaluating the performance of the DL models which are defined as

1 $$ {\text{Accuracy}} = (TP + TN)/(TP + FP + TN + FN) $$

2 $$\begin{aligned} \text {Precision}&= (TP)/(TP + FP),&\end{aligned}$$

3 $$\begin{aligned} \text {Recall}&= (TP)/(TP + FN),&\end{aligned}$$

4 $$\begin{aligned} \text {F1-score}&=(2/(\text {Precision}^{-1}+\text {Recall}^{-1}))&\nonumber \\&= (TP)/(TP+0.5(FP+FN)),&\end{aligned}$$

where *TP*, *TN*, *FP* and *FN*, respectively, denote the number of true positives, true negatives, false positives and false negatives obtained.

**Figure 7:**
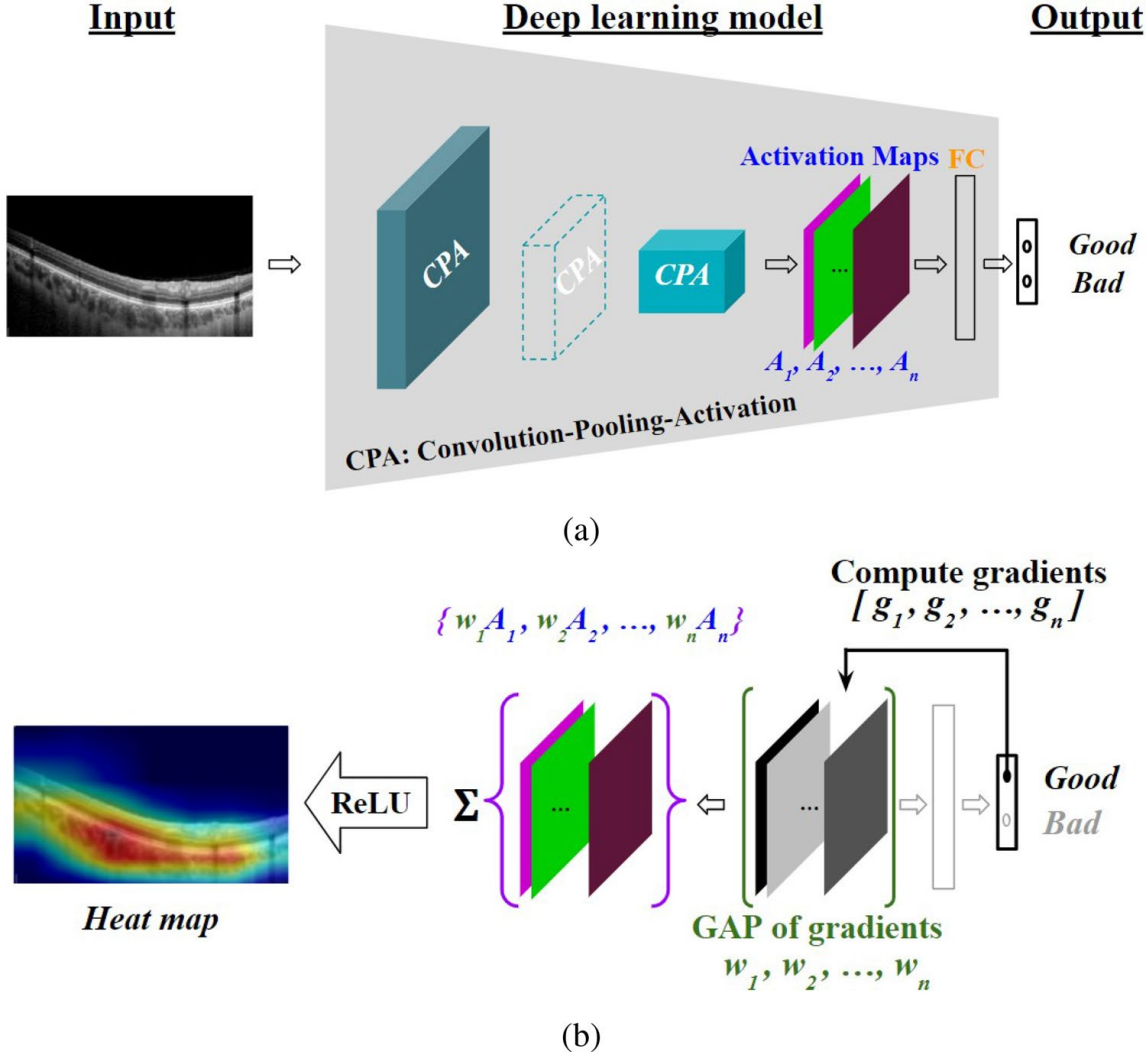
Schematic illustration of generating class-specific (Good) Grad-CAM output on a trained architecture/model.

#### Stratified *K*-fold cross validation

To obtain a mean performance of the models on different training data subsets, we perform stratified *K*-fold cross-validation. In particular, the dataset is randomly partitioned into $K (= 5)$ folds preserving the class ratios, and each fold is successively used as a test set while considering the union of the remaining $K -1 (= 4)$ as the training set. Finally, the average performance on the test set over *K*-folds is reported as model performance.

#### Visual explanations

At their inception, inner workings of high-performance DL models, owing to their complex architecture (consisting of convolution blocks, activation maps, FC layers and other components as shown in Fig. 7a), were not amenable to human intuition, and the outcomes could not be authenticated^48^. Machine-generated explanations, such as those generated by the ubiquitous gradient-based Grad-CAM technique^39^, have begun to overcome the aforementioned limitation. Specifically, as shown in Fig. 7b, gradients $g_{1}, g_2,...,g_n$ (*n* being the number of activation maps) corresponding to a specific class (‘good’, in the illustration) were computed with respect to respective activation maps $A_{1}, A_2,..., A_n$ of the final convolution layer. Importance weights $w_{1}, w_2,...,w_n$ corresponding to the activation maps are obtained via global average pooling (GAP) of the gradients. Subsequently, the weighted sum $\sum _{k} w_{k} A_{k}$ was computed and passed through the ReLU function ($\max (0,x)$ in variable *x*) to take only positive correlations into account. The resulting map is finally up-sampled to the input image size, and overlaid on the input image as a heat-map of explanation, with ‘hotter’ shades indicating higher relevance.

#### Subjective evaluation of visual explanations

**Figure 8:**
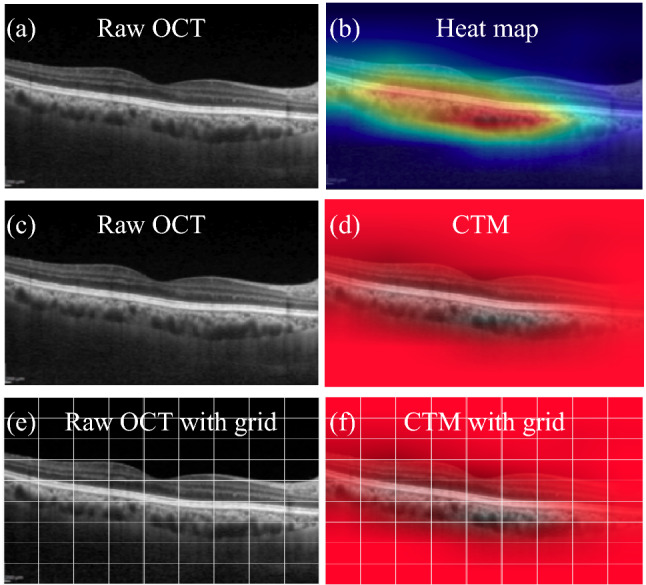
Visual explanations:Top row represents OCT with corresponding Grad-CAM generated heatmap; Middle row represents OCT with corresponding CTM; Last row represents OCT with corresponding CTM with grid on.

We propose to perform subjective scoring on the Grad-CAM visualizations to objectively evaluate the extent of localization of the choroidal layer by the DL models under consideration while determining the quality. However, in the usual Grad-CAM generated heatmaps, the interest region underneath the hot (focus) areas gets occluded making it difficult for the grader to access it. For each pixel in the OCT image, Grad-CAM generates a value ($g_c$) between 0 to 1, respectively, representing ‘low’ to ‘high’ relevant regions of the OCT for the deep learning model. In view of this, for a representative OCT image (shown in the Fig. 8a,c), we generated the color transparency map (CTM) (as shown in the Fig. 8d) using the corresponding Grad-CAM based heatmap (see Fig. 8b). In particular,to obtain CTM, we first generated a red channel mask where the red channel value is taken as 1 -$g_c$. Subsequently, we modified the raw OCT image by multiplying each of its intensity with its corresponding Grad-CAM value and converted the resultant image into a three channel (Red-Green-Blue) color image. Finally, we appended the red channel mask to the modified raw OCT image to generate the CTM. Such a map facilitates the grader to clearly visualize the structures relevant to the model’s decision-making. Based on these CTMs we designed the subjective grading strategy. Specifically, we proposed two scores, namely, overall choroid coverage (OCC) and choroid coverage within the visible region (CCVR). OCC is a relative score defined as the ratio between the visible choroid region in the CTM and the actual choroid region in the raw OCT image. On the other hand, the CCVR score is computed solely based on only the transparent region of CTM by taking the ratio of visible choroid region to the total visible region. Mathematically, OCC and CCVR can be written as

5 $$  OCC = (T \cap C)/C  $$

6 $$\begin{aligned} \text {CCVR}&= (T \cap C)/T,&\end{aligned}$$

where *T* and *C* denote the transparent region in the CTM and choroid region in the raw-OCT image, respectively.

In this setting, to facilitate graders performing the subjective grading, we developed a web-based user interface (UI) which displays the images and the list of parameters with respective scoring boxes to grade. Specifically, for each instance, grader is shown two pairs of images that constitute the raw OCT with corresponding CTM (see Fig. 8c,d) and the raw OCT image with corresponding CTM with an overlaid grid(see Fig. 8e,f). The second pair of images with overlaid grids are provided to further assist the grader in cases of any difficulty in comparing areas based only raw OCT image and its corresponding CTM (Fig. 8c,d). The UI is designed to have unique user credentials for graders to maintain between graders. As part of subjective analysis, we considered two graders for the current task. Further, we considered a subset of 60 OCT images from the dataset for grading the CTMs across three models. Finally, the reliability of grading among the graders were evaluated based on the Bland-Altman correlation score between the two graders given by $\sum _{i=1}^{N} (x_{i}y_{i})/ (\sqrt{(}\sum _{i=1}^{N} (x_{i})\sum _{i=1}^{N} (y_{i})))$, where $x_{i}$ and $y_{i}$ correspond to the scores given by two graders^49^.

## Data Availability

The dataset considered in the current study is part of an ongoing work and hence can not be made publicly available. However, the dataset is available from the corresponding author on reasonable request.
